# A Randomized, Open, Single-Centre, Crossed Study of the Effect of Food on the Pharmacokinetics of One Oral Dose of Alflutinib Mesylate Tablets (AST2818) in Healthy Male Subjects

**DOI:** 10.22037/ijpr.2020.113112.14116

**Published:** 2020

**Authors:** Songlin Zhu, Jun Deng, Qi Tang, Jianfu Heng, Jingjing Qu, Yong Chen, Xue Chen, Nong Yang, Xiaobao Liu, Kunyan Li

**Affiliations:** a *Department of Clinical Pharmaceutical Research Institution, Hunan Cancer Hospital, Affiliated Tumor Hospital of Xiangya Medical School of Central South University, Changsha, Hunan, 410006, China. *; b *Department of Early Clinical Trail Center, Hunan Cancer Hospital, Affiliated Tumor Hospital of Xiangya Medical School of Central South University, Changsha, Hunan, 410006, China.*; c *Department of Lung Cancer and Gastroenterology, Hunan Cancer Hospital, Affiliated Tumor Hospital of Xiangya Medical School of Central South University, Changsha, Hunan, 410006, China.*; 1 * S. Z. and J. D. contributed equally to this work.*

**Keywords:** Alflutinib mesylate tablets, Pharmacokinetics, Healthy volunteers, Bioavailability, High-fat meal

## Abstract

The aim of the study was to study the PK of AST2818 tablets after one oral dose in healthy male subjects on an empty stomach and in a postprandial state and to evaluate the effect of food on AST2818 bioavailability. Sixteen healthy Chinese male subjects were randomly divided into two groups: a fasting-postprandial group and a postprandial-fasting group. The drug was administered once per evaluation at a dose of 80 mg, with an interval of 22 days between the two treatments. The LC-MS/MS method was used to determine the concentrations of AST2818 and its metabolite AST5902. Plasma pharmacokinetic parameters were calculated by noncompartmental analysis (NCA). WinNonlin® version 7.0 was used to analyse PK parameters, and SAS version 9.4 was used for statistical analyses. After a meal, the peak concentration of alflutinib increased by approximately 53%, and the AUC increased by approximately 32%; The peak concentration of its metabolite AST5902 decreased by approximately 20%, and the AUC decreased by approximately 8%. There was no significant change in peak time. The peak AST5902 concentration and AUC_0-∞_ were 27.4% and 71.4%, respectively, of that of alflutinib. None of the subjects experienced serious AEs, and both fasting and high-fat meal administration were safe. There was no statistically significant difference between groups in AEs (*P* = 0.102, RR = 1.40) or adverse reactions (*P* = 0.180, RR = 1.30). The effects of food may not need to be considered for the clinical use of alflutinib. No serious AEs occurred, and drug administration was safe and tolerable after fasting or a high-fat meal.

## Introduction

Among malignant tumours, lung cancer has become the fastest growing disease, with serious negative consequences on human health and life. According to research data published in CA: A Cancer Journal for Clinicians, there were 1.8 million new cases of lung cancer worldwide in 2015, accounting for 13% of all new cases of cancer (1). Among all types of lung cancer, non-small cell lung cancer (NSCLC) is the most common, accounting for 75%~85% of all lung cancers. Epidermal growth factor receptor (EGFR) is a common driver gene of NSCLC, and EGFR mutations are found in approximately 10% of Caucasians and 30-40% of East Asians with NSCLC (2, 3).

Molecular targeted therapy has developed rapidly in recent years. Compared with traditional chemotherapy, which generally lacks tumour target specificity, the small molecule targeted tyrosine kinase inhibitors (TKIs) have an improved curative effect and fewer adverse reactions. EGFR-TKIs have become an important treatment for cancer and there are currently three generations of EGFR-TKIs (4, 5). Third-generation EGFR-TKIs are selective and irreversible TKIs designed specifically for EGFR mutations, such as EGFR/T790M. These inhibitors display weak inhibition of wild-type EGFR but greatly enhanced inhibitory activity against EGFR/T790M. Therefore, these TKIs show the greatest selectivity possible in inhibiting mutant EGFR/T790M activity without affecting normal EGFR activity. Currently, osimertinib (AstraZeneca) is marketed in the United States and China for patients with the EGFR drug-resistant mutation EGFR/T790M. The phase I AURA18 study (NCT02529995) assessed osimertinib pharmacokinetics (PK) in Chinese patients with advanced NSCLC who progressed following prior EGFR-TKI therapy. The results showed that osimertinib PK in the AURA18 Chinese patient population was consistent with that in the global population and supported 80 mg once-daily dosing (6-8). Hangzhou Eisen’s avitinib has completed major clinical studies and will be soon on the market. The alflutinib mesylate tablet (AST2818) is a third-generation EGFR-TKI independently developed by Shanghai Allist Pharmaceutical Technology Co., Ltd., in China as a new class I drug. Clinically, it is intended to treat lung cancer patients with the EGFR/T790M drug-resistant mutation, which have undergone a phase III clinical trial in NSCLC in China (http://www.chinadrugtrials.org.cn/; Registration number: CTR20182519). Early studies on the pharmacology and toxicology of AST2818 showed that it could effectively suppress the drug-resistant EGFR/T790M mutation with good tolerability. The stage I dose escalation study showed good tolerability of 20 mg, 40 mg, 80 mg, 160 mg, and 240 mg AST2818 (NCT02973763). The phase I/II study showed that the objective response rate (ORR) was 50% for 20 mg AST2818 and was greater than 65% for 40-160 mg AST2818. AST2818 showed good efficacy and safety at a dose of 80 mg.

Most small molecule targeted drugs are oral preparations, and thus, their bioavailability is readily affected by food than that of intravenous drugs (9-10). The bioavailability of small molecule TKIs, such as erlotinib, icotinib, afatinib, and sorafenib, is known to be affected by food (11-14). Therefore, in clinical practice, we should consider the influence of food on small molecule targeted drugs to avoid reducing drug efficacy or increasing adverse reactions.

The aim of this study was to investigate the bioequivalence of AST2818 and its metabolite AST5902 in terms of PK properties, safety and immunogenicity, in healthy Chinese adult males to provide clinical treatment guidance.

## Experimental

*Subjects*

We used a randomized grouping, balanced, single-dose, two-group (fed and fasted states), double-cycle and double-order crossover design to evaluate the effect of food on the bioavailability and the postprandial bioequivalence according to the technical guiding principles issued by the China Food and Drug Administration. This clinical research fully complied with the Declaration of Helsinki and Good Clinical Practice guidance. The study is registered in the Chinese National Registry (http://www.cde.org.cn/) under code CTR20182048. The clinical trial protocol and written informed consent form used in this study were both approved by the Medical Ethics Committee of Hunan Cancer Hospital. A total of 16 healthy male subjects were entered into this PK study after written informed consent was obtained. Before enrolment, the eligibility of each subject was determined at a screening visit at which a physical examination (including of blood pressure), electrocardiography, X-ray imaging and routine laboratory tests were performed. Subjects with a history of hypersensitivity to components of EGFR-TKIs or other serious diseases were excluded.

*Study Design and Treatments*

All patients were randomly divided into two study arms and received 80 mg alflutinib mesylate tablets (AST2818): arm A was treated on an empty stomach with no food for at least 10 h on the night of day -1 and no breakfast on day 0, and arm B was treated after no food for at least 10 h on the night of day -1 and a high-calorie, high-fat breakfast (943.46 kcal) on day 0. The high-fat meal consisted of two slices of whole wheat bread (100 g), one slice of ham (100 g), one slice of young Dutch cheese (40 g), 250 mL of whole milk, and 40 mg of pine nuts. The drug was administered once a cycle at an interval of 22 days. In the second cycle, the two arms were crossed. All subjects were instructed not to eat for at least 4 h after taking the drug.

*Measurement of Serum Drug Concentrations*

Blood samples were collected to evaluate the plasma concentration of AST2818 and its metabolite AST5902 within 30 min before drug intake and at 0.5, 1, 2, 3, 4, 6, 8, 10, 12, 24, 48, 72, 120, 168, 216, 336 and 504 h after drug intake. The blood samples (4 mL) were collected from the brachial vein and placed in an anticoagulant tube with ethylenediaminetetraacetic acid (EDTA) and then centrifuged at 2000 ×*g* for 10 min. The resulting serum samples were stored at -80 °C. In this study, the plasma concentrations of AST2818 and its metabolite AST5902 were determined using the liquid chromatography-tandem mass spectrometry (LC-MS/MS) method (15). The linear range of the plasma AST2818 concentration was 0.200-100.000 ng/mL, and the lower limit of quantification was 0.200 ng/mL. The linear range of the plasma AST5902 concentration was 0.0500~25.000 ng/mL, and the lower limit of quantification was 0.050 ng/mL.

*Pharmacokinetics Analyses*

The serum drug concentration versus time data were used to calculate the following parameters in each subject: C_max_, T_max_, AUC_0-t_, AUC_0-∞_, mean residence time from time 0 to the last measurable value (MRT_0–t_), and terminal half-life (t_½_). C_max_ was recorded as the peak value observed in each subject. AUC_0-t_ was estimated using the trapezoidal rule. The t_½_ was determined by fitting a linear regression to the drug concentration time curve. PK analyses were performed using WinNonlin® software, version 7.0 (Certara, L.P., Princeton, NJ, USA). PK parameters are expressed as the mean (standard deviation) or mean (range). The bioequivalence of AST2818 was accepted if the 90% confidence interval (CI) of the difference in the mean log-transformed value (test reference) was within the range of log (0.80) to log (1.25), which was established based on the Guidelines for Bioequivalence Studies of Generic Products (16).

*Safety Assessments*

Potential adverse events (AEs) were defined as those that occurred any time after the initial administration of study drug. AEs classified as possibly or definitely related to the study drug were listed as adverse drug reactions (ADRs). AEs were graded according to the National Cancer Institute Common Terminology Criteria for Adverse Events version 4.0.

## Results

*Baseline characteristics of the subjects*

In this study, a total of 16 healthy male adult subjects were randomly enrolled. The age, weight, BMI, smoking status, and drinking status of the 16 subjects were all in line with the inclusion criteria. The subjects’ demographic information and baseline characteristics are shown in Table 1.

*Plasma drug concentration time curve*

**Figure 1:**
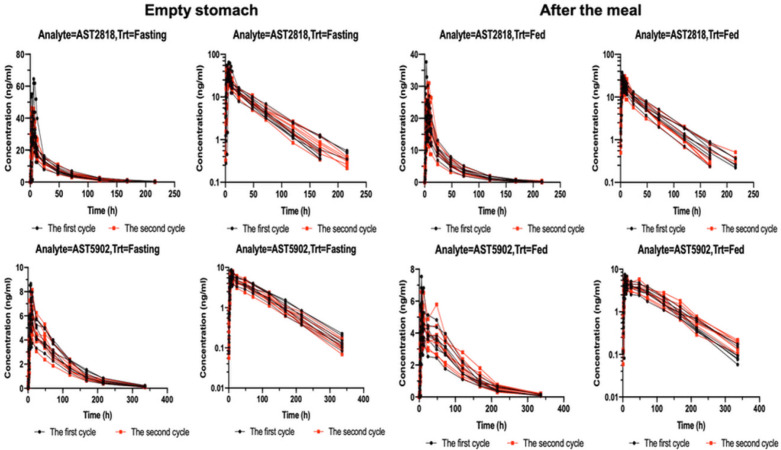
Concentrations of serum AST2818 and its metabolite AST5902 over time after a single oral dose of 80 mg alflutinib mesylate in the fast or postprandial state

Figure 1 shows the serum concentrations of AST2818 and its metabolite AST5902 versus time after dosing. The serum AST2818 concentration was below the lower limit of quantitative detection at 336 h after a single oral dose of 80 mg alflutinib mesylate in the fasted or postprandial state. Moreover, the serum metabolite AST5902 concentration was below the lower limit of quantitative detection at 504 h.

*PK analysis of AST2818 and its metabolite AST5902*

After healthy subjects were given a single dose of AST2818 on an empty stomach, the serum AST2818 concentration peaked at 6.0 h (median), the plasma half-life was 34.9 h, C_max_ was 25.4 ng/mL, and AUC_0-∞_ was 843.0 h × ng/mL. The serum concentration of the metabolite AST5902 peaked at 10.0 h (median), the plasma half-life was 57.7 h, C_max_ was 6.58 ng/mL, and AUC_0-∞_ was 572.0 h × ng/mL. These data are shown in Table 2.

After healthy subjects received a single dose of AST2818 in the postprandial state, the serum AST2818 concentration peaked at 6.0 h (median), the plasma half-life was 34.1 h, C_max_ was 39.6 ng/mL, and AUC_0-∞ _was 1110.0 h × ng/mL. The serum concentration (AST5902) peaked at 8.0 h (median), the plasma half-life was 58.6 h, C_max_ was 5.31 ng/mL, and AUC_0-∞_ was 529.0 h × ng/mL. These data are shown in Table 3. The main PK parameters of the geometric least square means, ratio and 90% CI of AST2818 and AST5902 are shown in Table 4.

The results showed no significant change in the peak time of AST2818 after a meal or on an empty stomach. Compared to the fasted state, the fed state showed a 53% increase in the peak concentration and a 32% increase in the AUC. Moreover, the peak concentration of the metabolite AST5902 decreased by approximately 20%, and the AUC decreased by approximately 8%.

*Safety assessment*

After drug intake on an empty stomach, a total of 14 subjects experienced 47 AEs, with an incidence of 87.5%. Most of the AEs were grade 1 or 2; there were no grade 4 or 5 events. Grade 3 AEs (increased myocardial enzymes) occurred in two (12.5%) subjects. No AEs were serious or led to study withdrawal.

After postprandial drug intake, a total of 10 subjects experienced 38 AEs, with an incidence of 62.5%. Most of the AEs were grade 1 or 2; there were no grade 4 or 5 events. Grade 3 AEs (increased myocardial enzymes) occurred in one (6.3%) subject. No AEs were serious or led to study withdrawal. The specific AEs are shown in Table 5.

## Discussion

This was the first single-dose PK study of alflutinib mesylate in healthy male subjects, and the effect of a high-fat diet on alflutinib absorption was also evaluated. According to the PK results of this preliminary clinical study, the sufficient sampling time enabled a description of the PK behaviour of alflutinib mesylate tablets after oral administration. The results showed that after healthy subjects were given a single dose of AST2818 on an empty stomach, the serum AST2818 concentration peaked at 6.0 h (median) with a plasma half-life of 34.9 h, and the plasma AST5902 concentration peaked at 10 h with a plasma half-life of 57.7 h.

A previous phase I/II clinical trial established a complete set of LC-MS bioanalytical methods for PK analyses of alflutinib and AST5902. The total chromatographic run time is 2.1 min, and a fast sample pretreatment method is achieved by using acetonitrile as a precipitating reagent. The lower limits of quantitation of alflutinib and AST5902 are 0.20 and 0.050 ng/mL, respectively, and the corresponding linear ranges are 0.20–100 and 0.050–25.0 ng·mL^−1^. This analytical method was successfully used for the PK analysis of alflutinib and AST5902 in human plasma (15). In the primary two-stage study, advanced NSCLC patients were given alflutinib mesylate at a single fasting dose of 80 mg; the peak alflutinib concentration was 45.3 ng/mL in stage one (trial no. ALSC001AST2818, N = 3) and 30.1 ng/mL in stage two (trial no. ALSC002AST2818, N = 8), and the peak AST5902 concentration was 7.68 ng/mL in stage one (trial no. ALSC001AST2818, N = 3) and 6.07 ng/mL in stage two (study no. ALSC002AST2818, N = 8). In this study, we used the LC-MS/MS method to evaluate the PK of alflutinib and AST5902 in healthy male subjects. Healthy subjects in this study were given the same dose of the experimental drug, and the peak concentrations of alflutinib and its metabolite AST5902 were 25.4 ng/mL and 6.58 ng/mL, respectively. Perhaps the different disease status between the healthy subjects and the NSCLC patients contributed to slightly higher peak concentrations in the cancer patients (17).

This study found that after a meal, drug absorption increased; the peak concentration of alflutinib increased by approximately 53%, and the AUC increased by approximately 32%. However, the peak concentration of its metabolite AST5902 decreased by approximately 20%, and the AUC decreased by approximately 8%. Whether this PK change has clinical significance is unclear. However, from the perspective of clinical safety, in the early stage of the dose escalation study, none of the subjects experienced dose-limiting toxicity, and the AEs were mild or moderate at doses of 20 mg, 40 mg, 80 mg, 160 mg, and 240 mg alflutinib. Therefore, although exposure to AST2818 increased after a high-fat meal, this may indicate a first-order process that does not affect drug safety. In addition, alflutinib and its metabolite AST5902 have similar pharmacological activities, and the proto-drug and its metabolite have similar plasma exposure when a patient reaches steady-state after continuous administration, wherein postprandial administration has little effect on metabolite exposure. Therefore, changes in exposure to the proto-drug and metabolite after a meal may not result in changes in efficacy or safety. Therefore, the effects of food may not need to be considered for the clinical use of alflutinib.

Regarding safety, the healthy male subjects given 80 mg alflutinib mesylate in tablet form (2 tablets) on an empty stomach or after a meal reported no serious AEs, and no AEs led to withdrawal or death (*i.e.*, the drug was safe and tolerated). The* P*-value of AEs associated with taking the drug on an empty stomach versus taking it after a meal was 0.103, and the relative risk (RR) was 1.40. The P-value of adverse reactions associated with taking the drug on an empty stomach versus taking it after a meal was 0.180, and the RR was 1.30.

**Table 1:** Characteristics of the subjects (n = 16).

**Item**	**Subgroup**	**Value**
Age (years), mean ± SD		30.72 ± 6.89
Sex, n (%)	Male	16 (100%)
	Female	0 (0%)
Body mass index (BMI, kg/m^2^), mean ± SD		22.11 ± 1.78
Smoking status, n (%)	Yes	0 (0%)
	No	16 (100%)
Allergy to drugs/food/other substances, n (%)	Yes	0 (0%)
	No	16 (100%)

**Table 2:** Pharmacokinetic parameters of AST2818 after a single oral dose in healthy subjects in the fasted or postprandial state (n = 16).

	**Pharmacokinetic parameter**	**Units**	**Mean ± SD**	**CV (%)**
Fasted	*λ*_z_	1/h	0.020 ± 0.003	14.800
	t_1/2z_	h	34.90 ± 5.32	15.20
	^*^T_max_	h	6.00 (2.00-8.00)	
	C_max_	ng/mL	25.40 ± 5.61	22.10
	AUC_0-t_	h × ng/mL	827.00 ± 196.00	23.70
	AUC_0-∞_	h × ng/mL	843.00 ± 200.00	23.70
Postprandial	*λ*_z_	1/h	0.021 ± 0.002	12.100
	t_1/2z_	h	34.10 ± 4.20	12.30
	^*^T_max_	h	6.00 (3.00-8.00)	
	C_max_	ng/mL	39.60 ± 12.30	30.90
	AUC_0-t_	h × ng/mL	1090.00 ± 242.00	22.00
	AUC_0-∞_	h × ng/mL	1110.00 ± 245.00	22.00

^*^Median (Min-Max).

**Table 3:** Pharmacokinetic parameters of AST5902 after a single oral dose in healthy subjects in the fasted or postprandial state (n = 16).

	**Pharmacokinetic parameter**	**Units**	**Mean ± SD**	**CV (%)**
Fasted	*λ*_z_	1/h	0.012 ± 0.001	8.200
	t_1/2z_	h	57.70 ± 4.72	8.20
	^*^T_max_	h	10.00 (6.00-12.00)	
	C_max_	ng/mL	6.58 ± 1.24	18.80
	AUC_0-t_	h × ng/mL	561.00 ± 114.00	20.30
	AUC_0-∞_	h × ng/mL	572.00 ± 117.00	20.50
Postprandial	*λ*_z_	1/h	0.012 ± 0.002	14.300
	t_1/2z_	h	58.60 ± 8.71	14.90
	^*^T_max_	h	8.00 (6.00-24.00)	
	C_max_	ng/mL	5.31 ± 1.22	22.90
	AUC_0-t_	h × ng/mL	517.00 ± 105.00	22.30
	AUC_0-∞_	h × ng/mL	529.00 ± 107.00	20.30

^*^Median (Min-Max).

**Table 4:** Main pharmacokinetic parameters of the ratio of the geometric least square means and 90% CI of AST2818 and AST5902

**Analyte**	**Parameter**	**Geo LSM - Fed**	**Geo LSM - Fasted**	**Ratio (%) Fed/Fasted**	**90%CI**
AST2818	AUC_0-∞_	1086.85	820.48	132.47	125.82-139.47
	AUC_0-t_	1067.18	804.41	132.67	125.97-139.72
	C_max_	37.99	24.75	153.47	137.89-170.81
AST5902	AUC_0-∞_	518.44	560.61	92.48	90.65-94.34
	AUC_0-t_	506.67	550.07	92.11	90.13-94.13
	C_max_	5.18	6.46	80.21	74.52-86.34

**Table 5:** Summary of adverse events after drug administration (n = 16).

**System organ class and preferred term**	**Empty stomach**		**Postprandial**		**Relative risk**
	**Events (n)**	**Incidence (%)**	**Events (n)**	**Incidence (%)**	
Increased myocardial enzymes	15	9 (56.30%)	8	2 (12.50%)	4.50
Decreased blood pressure	11	7 (43.80%)	9	6 (37.50%)	1.17
Elevated blood sugar	3	3 (18.80%)	5	5 (31.30%)	0.60
Increased total bile	3	2 (12.50%)	1	1 (6.30%)	2.00
Increased blood bilirubin	2	2 (12.50%)	0	0	-
Increased serum myoglobin	1	1 (6.30%)	1	1 (6.30%)	1.00
Elevated serum uric acid	1	1 (6.30%)	1	1 (6.30%)	1.00
Decreased white blood cell count	1	1 (6.30%)	0	0	-
ST segment elevation in the electrocardiogram	1	1 (6.30%)	0	0	-
Decreased neutrophil count	1	1 (6.30%)	0	0	-
Elevated white blood cell count	0	0	1	1 (6.30%)	0
Increased alanine aminotransferase	0	0	1	1 (6.30%)	0
Elevated red blood cell count	0	0	1	1 (6.30%)	0
Combined with elevated bilirubin	0	0	1	1 (6.30%)	0
Haemoglobin elevation	0	0	1	1 (6.30%)	0
Elevated platelet count	0	0	1	1 (6.30%)	0
Increased neutrophil count	0	0	1	1 (6.30%)	0
Sinus bradycardia	7	6 (37.50%)	3	2 (12.50%)	3.00
Haematuria	0	0	2	2 (12.50%)	0

## Conclusion

Changes in proto-drug and metabolite exposure induced by the postprandial administration of alflutinib mesylate tablets (80 mg) may not result in changes in efficacy or safety. Therefore, the effects of food may not need to be considered for the clinical use of alflutinib.
